# Sustaining the integrity of the threatened self: A cluster-randomised trial among social assistance applicants in the Netherlands

**DOI:** 10.1371/journal.pone.0252268

**Published:** 2021-06-03

**Authors:** Mira Bierbaum, Eleonora E. M. Nillesen

**Affiliations:** 1 UNU-MERIT, Maastricht, the Netherlands; 2 Maastricht Graduate School of Governance, Maastricht University, Maastricht, the Netherlands; 3 International Labour Organization, Geneva, Switzerland; University of York, UNITED KINGDOM

## Abstract

Stereotypes and stigma associated with living on welfare or a low income can be a psychological threat that hampers performance and undermines aspirations. Our paper explores the potential of a novel self-affirmation intervention to mitigate such adverse impacts. The intervention comprises a verbal self-affirmation exercise for applicants during their first meeting with a caseworker. We conduct a cluster-randomised trial among a sample of 174 applicants for social assistance benefits in a Social Services office in Maastricht, the Netherlands. We measure outcomes on feelings of self-worth, stress, societal belonging, job search behaviour self-efficacy and cognitive performance immediately after the meeting. In our full sample, the intervention has a negative impact on feelings of societal belonging, but no effect on other outcomes. Effects, however, vary by subgroups. Our treatment increases negative feelings of self-worth and negatively affects societal belonging, but also improves cognitive performance among the group that had paid work in the previous two years. By contrast, self-affirmation positively impacts job search behaviour self-efficacy and cognitive performance for individuals who face increased challenges to (re)integrate into the labour market, proxied by lower levels of education or social assistance receipt in the previous two years. Since our intervention gives rise to testing more than one null hypothesis, we control the false discovery rate using the Benjamini-Hochberg approach. Our findings are sobering. Effects only remain significant for negative feelings of self-worth and improved cognitive performance for one particular subgroup: individuals with paid work in the past two years. This suggests self-affirmation may have reminded them of the time they still had a job, hence creating a backlash effect on feelings of self-worth. At the same time, they may have felt a need to distinguish themselves from others on social assistance benefits resulting in better cognitive performance. These interpretations are consistent with theory and empirical evidence on social identity and self-categorisation. We discuss the implications of our results and outline avenues for future work.

## Introduction

The threat of being judged or treated negatively in light of stereotypes is a situational predicament termed “stereotype threat” [1–3]. Members of stigmatised groups feel at risk that their behaviour is assessed against the backdrop of these stereotypes. Stereotype threat can induce a disruptive state, hampering self-esteem, cognitive performance, effort levels, and undermining aspirations [4, 5]. Individuals may thereby be more or less susceptible to feelings of stigma depending on their personal history and background [6–8].

People living in poverty or receiving welfare benefits find themselves regularly confronted with stigmatising terms such as welfare dependency and underclass, or categories of moral worth such as “the undeserving poor”. Living on a low income and claiming benefits has been portrayed in stereotypical ways in the media or the political discourse, referring to characteristics or attitudes such as being lazy, incompetent, or free-riding on society [9–11]. The literature on welfare stigma demonstrates its prevalence and persistence across many societies, for example in the United States, United Kingdom, Norway, or the Netherlands [9, 10, 12–21].

Indeed, welfare stigma has been well documented and may originate from negative social attitudes instigated by what Besley and Coate [22] refer to as statistical discrimination. That is, society values certain characteristics like willingness to work and independence, and perceives welfare recipients to possess on average less of these desirable characteristics than non-claimants. An alternative explanation is taxpayer’s resentment [22]. Taxpayers may have stigmatised perceptions of recipients as opportunistic free-riders who compete for and exploit scarce societal resources [22, 23] in cases where welfare is tax-financed, such as social assistance benefits. Such perceptions may elicit emotions of contempt and disgust and can result in active (harassment) and/or passive (neglect, exclusion) harmful behaviour against this group [24].

Consequences of welfare stigma can be dire. Contini and Richiardi [25] show theoretically how welfare stigma can act as a deterrence to benefit take-up as individuals anticipate lower employability. Yet, it may also lead to welfare entrapment once benefits are received due to negative psychological consequences, such as discouragement that affects job search intensity and employment probability. Empirical research demonstrates that decisions to completely refrain from using or applying for social benefits can indeed be utility-maximising due to stigma, leading to significant levels of non-take-up [26–29].

However, living on a low income and welfare receipt is associated with many more socio-economic and psychological consequences than stigma alone. Scarce resources, stress, and low social standing are also likely to play a role. Over the past decades, evidence has accumulated that these conditions are associated with distinct patterns of how individuals think, behave, or navigate their lives [7, 30–33]. Living on a low income is associated with lower feelings of self-worth, less favourable perceptions of self-efficacy, avoidance-based behaviour, a lower sense of societal belonging and withdrawal from public activities [31, 34]. Apart from seriously affecting well-being through the psychological and relational aspects of reduced feelings of self-worth and social inclusion, this can lead to biased information processing, reduced cognitive performance and low self-efficacy beliefs that inhibit activities such as job search efforts.

Self-affirmation theory [35] has been proposed as a tool to maintain a global sense of personal integrity by affirming an important aspect of the self related to values or attributes not under threat, thereby curbing these adverse effects [36, 37]. In this paper we examine the effectiveness of a self-affirmation exercise for individuals who are unemployed, live on a low income, and are in the process of applying for social assistance benefits at a Social Services office in a cluster-randomised trial. We conduct our study in a novel setting where the potential impact of a stereotype threat is particularly high: a Social Services office where applicants for social assistance have to physically come and meet with caseworkers to receive income support and discuss their job search strategies. A visit to the Social Services office is arguably a situational cue that triggers a stereotype threat, both by raising concerns about being associated with stereotypes that have been internalised, thereby undermining self-identity, and for fear of being treated poorly (see [7, 38] for evidence and a discussion of this phenomenon). Our contributions are twofold. First, we link the literatures on self-affirmation and living in poverty and on welfare by using a self-affirmation exercise in the context of a Social Services office, a new domain that has not been explored previously. Second, we test the applicability of a low-cost, non-intrusive and easy-to-implement intervention to improve social and psychological impacts for social assistance applicants that ultimately may also affect labour market outcomes for this group.

Our findings are as follows. We find limited evidence of an average effect. Self-affirmed applicants report weaker feelings of societal belonging than non-affirmed individuals, but there is no impact on the other outcomes. Yet, self-affirmation may not affect everyone equally and in the same way, and social assistance applicants are a diverse population in terms of unemployment duration or education level. We therefore also report outcomes for relevant subgroups.

The heterogeneity analysis reveals that treatment causes a 0.3 standard deviation increase in negative feelings of self-worth among those who had no social assistance benefit in the past two years and 1.1 standard deviations for those who had paid work in the previous two years. Also, treatment reduces feelings of societal belonging by about 0.4 standard deviations especially among this group, suggesting that self-affirmation may act as a prime for the domain under threat rather than distracting from it. Treatment increases job search behaviour self-efficacy by about 0.5 standard deviations among respondents with lower levels of education. It also improves cognitive performance by 0.4 to 0.6 standard deviations for those who had paid work in the past two years as well as individuals who previously received social assistance benefits.

Our effects are sizeable but only two results hold up to correcting for multiple inference using the Benjamini-Hochberg procedure to control the false discovery rate (FDR): increased levels of negative feelings of self-worth and higher cognitive performance among people who had paid work in the previous two years. We believe that the self-affirmation may have unintentionally reminded them of their previous status as employee, thereby negatively affecting feelings of self-worth. Also, this group may then have a stronger wish to distinguish itself from other social assistance applicants by trying harder on cognitive performance tests—a phenomenon that has been observed in studies on social identity and self-categorisation. All in all, we provide the first empirical evidence on the role of self-affirmation in the realm of welfare assistance and come to the conclusion that self-affirmation may not work as intended in such a context.

The article proceeds as follows. In the next section we discuss relevant literature and develop a conceptual framework that details how self-affirmation may ‘work’ in the context of welfare and activation; section three describes the intervention and context; we present the empirical strategy in section four; section five comprises a discussion of the results and section sets out our conclusions.

## Conceptual framework

The basic premise of self-affirmation theory is that individuals have an innate need for maintaining the integrity and worth of the self. By affirming values that are important to them, individuals have more psychological resources available to view a potential threat to their integrity from a higher level of construal. This then helps to not let it affect their overall feeling of personal integrity to the same extent [39]. The self system thereby comprises different domains, including roles, for example as student or employee; values, such as religion; group identities based on race or nation; central beliefs, such as political beliefs; goals, for example academic achievements or achievements at work; and relationships [40].

A psychological threat occurs when a person perceives that the environment challenges her self-integrity. For instance, job loss can threaten someone’s role as a valued employee or reliable breadwinner. Such a threat sets in motion a number of interrelated mechanisms [41]: (1) a physiological stress response, (2) suppression processes in order to avoid negative thoughts and emotions, and (3) increased monitoring processes of the environment in relation to oneself. Together, these three mechanisms consume mental resources, and the increased cognitive load has negative repercussions on working memory, cognitive performance and problem-solving abilities. Self-affirmation is expected to mitigate these threats by restoring or maintaining an individual’s continued adequacy in face of a threat, and enable a more holistic perspective of the self.

Self-affirmation exercises differ in terms of affirmation domain (specific value or personal characteristic), attainment of this value or characteristic (provided by the researcher or chosen by the respondent), and procedure (respond to a scale, write an essay, or imaginary techniques) [42]. A typical manipulation asks participants to rank a list of personal characteristics, values, or skills. Subsequently, participants write a brief essay in which they explain why the top-ranked value or characteristic is important to them. Participants in the non-affirmed condition reflect on a medium- or bottom-ranking value or characteristic [43, 44]. McQueen and Klein [42], Sherman and Cohen [40], Sherman [39] and Cohen [36] summarise the burgeoning literature on self-affirmation theory and interventions. Earlier lab experimental studies demonstrate that self-affirmation makes subjects less likely to change attitudes to reduce cognitive dissonance than non-affirmed subjects, by maintaining or restoring self-integrity [45]. Others use self-affirmation interventions to study whether self-affirmation attenuates biased information processing or affects emotions or behavioural intention when subjects are presented with self-threatening health information (e.g. the risk of smoking or drinking) [4, 46, 47]. In recent years, self-affirmation interventions have extended to other evaluative domains including education [48–50], the workplace [51, 52] and poverty [37, 53, 54]. While various self-affirmation interventions have reported positive outcomes, others found results to be heterogenous, mixed or sometimes even negative, depending on selected outcomes, individual differences, psychological factors and the social context [55–61].

Our contribution lies at the interface between the literature on self-affirmation theory and evidence on the one hand and the psychological effects of living on a low income and welfare stigma on the other hand. We investigate whether self-affirmation reduces stereotype threats and its consequences in the context of welfare, in particular social assistance schemes. The next paragraphs review the empirical literature on how self-affirmation may affect relevant outcomes in this context, namely sense of self-worth, stress, societal belonging, self-efficacy, and cognitive performance.

An individual’s sense of self-worth may be severely damaged by being deprived of a defining domain in one’s self system, such as being a valued employee or breadwinner. According to Critcher and Dunning’s [43] ‘affirmation as perspective’ model, the damaged identity may dominate one’s self-concept and narrow the scope towards the threatened domain, thereby letting it loom disproportionately large. As a result, self-evaluations are more contingent on the threatened domain than on a larger concept of the self. Self-affirmation has been shown to affect self-worth [62] and some evidence also suggests that self-affirmative messages can have a stress-buffering effect [55, 63, 64]. Moreover, feelings of social exclusion may be reduced through self-affirmation as it buttresses a person’s self-integrity [43, 65].

A large number of studies on self-affirmation explore its effects on self-efficacy. Perceived self-efficacy refers to “people’s beliefs about their capabilities to produce designated levels of performance that exercise influence over events that affect their lives” [66, p. 71]. Self-efficacy is a prominent variable in the job search literature and hence of particular importance in our context. Liu et al. [67] for example conduct a meta-analysis of 60 studies and conclude that job search interventions that included an element to bolster self-efficacy (such as enactive mastery of job search behaviours or verbal self-guidance) increased the odds of obtaining employment considerably.

Evidence of self-affirmation on self-efficacy exists, for instance, in the context of caffeine intake [47], healthy eating [68], or anti-smoking messages [69]. Affirmed participants had stronger beliefs in their abilities to adjust their behaviours in health-promoting ways; on average, outcome measures were at least half a standard deviation higher than in the control group. Such studies are typically based on the assumption that self-affirmation facilitates systematic and balanced processing of information, that affirmed participants are more open to information, and that they assess information and the role of the context and themselves more honestly –although see [58] for contrasting evidence. Alternatively, self-affirmation contributes to reducing stress, which, in turn, could enhance self-efficacy [68]. Or, self-affirmation affects self-efficacy through its effect on self-regard and perceptions of ability and control [69]. Self-efficacy is thus plausibly both a direct outcome and a possible mediating channel affecting some of the other outcomes. The distinction between job search behaviour self-efficacy and job search outcome self-efficacy is important in this context [70]. While job search behaviour is primarily controlled by the jobseeker, job search outcomes are the result of a multitude of interrelated factors, for instance educational attainment, profession, age, and the labour market situation. Hence, if self-affirmation allows for a more honest assessment of one’s circumstances and one’s own contributions, then it can be expected that self-affirmation enhances job search behaviour self-efficacy. In contrast, the effect is ambiguous regarding job search outcome efficacy, where less biased processing of information could lead to more positive or negative assessments depending on the individual situation in relation to the job market.

Finally, self-affirmation has been associated with changes in cognitive performance. Some evidence suggests that self-affirmative interventions reduce achievement gaps based on race or sex [2, 48, 49, 71]. Miyake et al. [49], for instance, find that two brief writing exercises (10 to 15 minutes) over a 15-week course increased women’s modal physics grade from the C to the B range and thereby reduced the gender achievement gap considerably. Affirmation was especially effective for women who believed that men do better on physics than women. Other research indicates that self-affirmation enhances problem-solving among chronically stressed individuals with impaired problem-solving capacity [72]. Hall et al. [37] conclude that self-affirmation had positive effects on cognitive performance (using Raven’s Standard Matrices) among participants recruited at an urban soup kitchen. For participants in the affirmed condition, the increase in cognitive performance was comparable to the difference in performance between an average 55-year-old and 45-year-old. The same study also demonstrated that an oral affirmation could increase openness to potential threatening information, echoing results from health-related studies. Affirmed participants were more likely to stop at a table where fliers on Earned Income Tax Credits and Volunteer Income Tax Assistance were offered, and to take a flier. Lastly, Banker and colleagues [54] show that affirmed participants—individuals living on a low income in a slum in Mumbai—were subsequently more likely to choose a more challenging task.

However, not all studies find strong and positive effects. Protzko and Aronson [60], for example, are unable to replicate large and positive effects of Cohen and colleagues [73] on reducing the academic achievement gap among Hispanic and Black students in schools where these negatively stereotyped groups comprise either the majority or minority. The impact of a self affirmation exercise on these outcomes thus seems to vary with the context and the population at hand turning it into an empirical question. We now turn to such an example.

## Intervention and context

We conduct our study in the Social Services office of the city of Maastricht, the Netherlands. Our aim was to develop an affirmation that could easily be included in the standard work process and implemented by caseworkers themselves. Applying for social assistance proceeds as follows. The first step is to submit an application electronically. Within two working days after submission, individuals have to appear in person at the Social Services office with a valid ID card for a short eligibility screening. The subsequent process depends on the applicant’s age. For applicants older than 27 years, an intake meeting is planned immediately. Applicants aged 18 to 27 have a compulsory job search period of four weeks during which they have to document their job search efforts before they have their intake meeting. Furthermore, some of these applications (based on the region) are processed by a caseworker of a separate team (team youth). Characteristics beyond the applicants’ age range (that is, 18–27 and above 27) are not taken into account when assigning applicants to caseworkers, making this essentially a random process.

During the intake meeting, applicants have to provide all necessary documents to check benefit eligibility. In addition, they are informed about their rights and obligations as social assistance recipients, including job search requirements. Our intervention takes place during the individuals’ intake meetings at the Social Services office. The intake meeting lasts approximately one hour and aims to get to know the applicant and informs them about their rights, obligations and job search activities. We hence believe that this meeting lends itself better to the inclusion of a self-affirmation intervention than the very first initial screening which is only a formal and quick procedure.

The verbal affirmation procedure we used was developed based on Cohen and Sherman [36], Hall et al. [37] and McQueen and Klein [42] as well as principles of motivational interviewing [74]. We developed a protocol stipulating that: (i) the caseworker should seek citizens’ permission to talk about personal values; (ii) the affirmation should be conducted at the beginning of the meeting; (iii) the self should be affirmed in a domain chosen by the participant that is outside the threat; and (iv) the affirmation should be introduced in descriptive and not in psychological terms.

The meeting would proceed as follows: The caseworker welcomed the applicant and then continued to introduce the study if the following eligibility criteria were met: Applicants had to be (i) older than 17 years and (ii) legally competent. Moreover, caseworkers did not introduce the study if (iii) language skills were seriously deficient, they had (iv) doubts about the individual’s mental capacity to provide informed consent, or if (v) the atmosphere was emotionally loaded or aggressive. Caseworkers were also asked to keep track of the cases when they decided against introducing the study and the reason for doing so. Unfortunately, this was not implemented in a consistent manner so that we have incomplete information on the majority of these cases.

If eligible, the caseworker introduced the study and explained its aims in very general terms, the paper-based survey participants were asked to fill in after the meeting, and the voucher of EUR 5 that participants would receive upon completion of the survey to thank them for their participation. Caseworkers also informed participants about the anonymous treatment of their personal survey data. Participants were then asked to sign a consent form they later returned with the survey. Caseworkers subsequently read out the following text to study participants in the treatment group:

As you know, the aims of this meeting are to [explain the objectives of the meeting]. But before we start with these topics, I would like to get an idea of who you are and what is important for you—not related to work, but to you as a person. Is that okay?

*[Participant answers. If he or she does not agree, the self-affirmation is not implemented.]*

Can you describe to me an experience or event that made feel you successful or proud?

*[Participant answers. If necessary, it is possible to give examples. For example: Some people tell about values they find important, for instance their religion. For others, certain activities or hobbies are important or they are proud of personal characteristics. Or they talk about relationships, for instance with their family or friends.]*

Can you explain why that made you feel proud/why that felt good?

*[Participant answers.]*

Participants in the non-affirmed (control) condition were asked neutral introductory questions for example about the mode of transport they used to get to the Social Services office.

We pre-tested the self-affirmation exercise with two caseworkers, one from the regular intake team and one from the team youth. Next, we trained caseworkers in a two-hour workshop that included a presentation on the intervention logic and hypotheses and various role plays.

We conducted the experiment between October 2017 and December 2018. This was a period characterised by economic growth and an improving labour market situation after the economic downturn following the 2007/2008 economic and financial crisis. The crisis had led to rising numbers of unemployment benefits and social assistance recipients. In 2017, the unemployment rate in Maastricht was still 5.6 per cent, and 4.7 per cent in the province of Limburg, roughly equal to the national average [75]. From May 2017 onwards the number of social assistance applicants decreased continuously. These developments had implications for the implementation of our study. Participants were recruited on a rolling basis and the improved economic and labour market situation meant that the average number of weekly intake meetings and hence potential participants was considerably lower than foreseen during the design phase of the experiment—an issue to which we return in the description of the sample.

## Research method

### Cluster-randomised trial

We use a cluster-randomised design with one treatment and one control arm. Social assistance applicants are randomly assigned to caseworkers, following standard organisational procedures of the Social Service office. Caseworkers therefore represent the clusters in which social assistance applicants are naturally grouped. We had 15 caseworkers that could be randomised into either the treatment or control group. Randomisation at the cluster level (the caseworkers) was preferred over randomisation at the individual level (the social assistance applicants) after weighing the trade-offs between statistical efficiency and the research setting [76–78]. We discuss these trade-offs in more detail below.

Clearly, randomisation at cluster level is costly. Statistical efficiency is lost as individuals within clusters tend to be more similar than individuals across clusters. The intracluster correlation coefficient (ICC) *ρ* quantifies this similarity. *ρ* is defined as the share of between-cluster variance $\sigma_{B}^{2}$ in the overall variance *σ*^2^ (the sum of between- and within-cluster variance $\sigma_{B}^{2}$ and $\sigma_{W}^{2}$). Three main reasons for having positive within-cluster correlation are (1) clustering of population characteristics; (2) variations in response to intervention; and (3) correlations due to interactions between participants [77]. The first—clustering of population characteristics—is unlikely to play a role in our setting. As new applicants are not systematically allocated to caseworkers there is no reason to believe that applicants’ characteristics differ systematically across caseworkers. Interactions among individuals is equally unproblematic as outcome variables are measured directly after the meeting and applicants who fill in the survey have no possibility to interact with other applicants in the meantime.

Variations in response to the intervention, however, are a likely source of within-cluster correlation. Different characteristics of caseworkers may be responsible for variations in responses across clusters. Possible distinguishing features that may interact with the intervention are a caseworker’s general understanding of and approach to her work (e.g. strong emphasis on rule implementation and fraud detection vs. a focus on supporting services or motivation). Caseworkers may also differ in their openness and commitment to experimenting with new methods and learning and their skills, flexibility and perceived “bandwidth” to conduct the self-affirmation as outlined in the protocol may vary. Finally, with a small number of clusters, randomisation may not ensure adequate balance between caseworkers in the treatment and control condition [77, 79]. The possibility that caseworkers are on average not similar regarding potentially confounding factors enhances the concern that applicants’ responses may to some extent vary depending on who implements the treatment. Finally, the small number of clusters also complicates data analysis although models for clustered data with very few clusters have recently attracted more attention and several options are available to adequately deal with this issue [80].

Consider individual-level randomisation as an alternative. Having caseworkers “switch” between treatment and control conditions could lead to caseworkers mixing them up [81, 82]. Karlan and Appel [83] also caution that researchers should not underestimate the mental effort and flexibility that it takes to implement even small changes with regard to familiar tasks and routines. Moreover, even if caseworkers are willing to deliver the treatment and control condition as planned and ostensibly do so, it is possible that the way they conduct meetings in the control group are unconsciously influenced by experiences they have had with the experimental group and the training in general [78]. Or, caseworkers that have positive experiences with the self-affirmation exercise may consider withholding this treatment from the control group as ethically wrong.

We are not able to assess the extent to which these situations actually occurred, nor control for them if they did. We therefore decided to opt for a cluster randomised design, following most experimental studies on activation in welfare organisations (see e.g. Malmberg-Heimonen and Tøge [84] or Behncke et al. [85]). We take the clustered nature of the data into account in both our sample size calculations and data analysis. Encouragingly, the ICC is low for all outcome variables, as reported in S1 Table. The size of all coefficients is very moderate and in most cases below the values assumed for the power calculations.

### Sample size and randomisation

The number of participants per caseworker is based on efficiency considerations. A rule of thumb is that further gains in power become modest as soon as the number of participants per cluster is larger than the reciprocal value of *ρ*. With an assumed ICC of 0.05, the optimal number of participants per caseworker is 20.

We used the *PowerUp!* tool for an ex ante power analysis calculating minimum detectable effect sizes and minimum required sample sizes [86]. We had 15 caseworkers participating in the study. We would have been able to detect effect sizes of approximately 0.49 standard deviations (*α* = 0.05; two-tailed test; 1-*β* = 0.80) with a sample of 300 individuals distributed among 15 clusters. Due to Dutch labour market improvements in the course of 2018, however, our final sample consisted of only 174 individuals, allowing us to detect effect sizes of approximately 0.57 standard deviations. This is in the same order of magnitude as effect sizes that other affirmation studies found on feelings of self-worth, self-efficacy, or cognitive performance.

Since several caseworkers only dealt with young applicants, restricted randomisation was used to ensure balance based on team affiliation [77]. We employed the *randomtreat* command [87] in Stata (version 15) to randomise caseworkers into the treatment and control condition, stratified by team affiliation (regular intake team and team youth). We set a seed for replicability. Seven caseworkers were distributed to the treatment group and eight caseworkers to the control group. Two caseworkers in the experimental and three caseworkers in the control condition exclusively worked with young people.

### Outcome measures

Outcomes are measured at the level of the participant. Directly after the intake meeting, participants were asked to fill out the survey in private. Our variables of interest were embedded in a paper-based survey on participants’ satisfaction with the intake meeting:

#### Self-worth

We measured self-worth with 14 items based on Critcher and Dunning [43]. Participants rated items on nine-point Likert scales ranging from 1 (do not agree at all) to 9 (agree completely). We created separate indices for positive and negative feelings of self-worth.

#### Stress

The stress measure was based on three items presented by Creswell et al. [55], but the wording was adjusted. Participants were asked to respond to statements on whether they experienced the meeting as stressful, difficult, or threatening. All items were measured on a five-point Likert scale that ranged from 1 (do not agree at all) to 5 (agree completely). We averaged the scores to create a composite measure of stress.

#### Societal belonging

**Fig 1:**
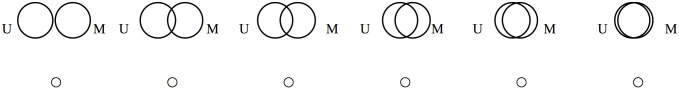
Adapted inclusion of community in self (ICS) scale. *Source*: Author’s own illustration based on Mashek et al. [88].

Societal belonging was measured on an ordinal, six-point scale that ranged from 1 (low) to 6 (high). We adapted the inclusion of community in self scale [88] which is a single-item cross-validated [89] pictorial measure that offers a simple account of community connectedness (see Fig 1). The measure consists of six pairs of circles. The first pair of circles does not overlap at all. In the following pairs, the overlap increases constantly until the sixth pair of circles nearly overlaps completely. We replaced ‘community’ by ‘society’ in our survey. Respondents received the explanation that in each image, the left circle represents themselves and the right circle refers to society. We broadly defined society as “all people together, and how they interact with each other”.

#### Job search behaviour self-efficacy

Our job search self-efficacy measure was based on Saks et al. [70] who explicitly distinguish between self-efficacy related to job search behaviour and outcomes. We left out some of the items and adjusted others to better reflect the context. We included nine items that referred to job search self-efficacy behaviour (for instance, belief that someone can prepare a good CV, plan weekly job search activities, or find appropriate vacancies). All items were measured on five-point Likert scales ranging from 1 (not confident at all) to 5 (completely confident). These questions were only included for participants who had no exemption from their job search requirements. Reasons for exemptions granted by the caseworker include, *inter alia*, work incapacity, certain social circumstances, provision of informal care, or caring for children younger than five.

#### Cognitive performance

Following Hall et al. [37], cognitive performance was measured using Raven’s Standard Progressive Matrices (RSPM). RSPM are considered a non-verbal estimate of fluid intelligence. We selected 12 out of 60 matrices based on Bilker et al. [90, Form A] who used abbreviated forms of the RSPM that are highly predictive of the complete 60-item RSPM scale.

#### Relevant covariates

We collected information on a number of relevant covariates including respondents’ sex, age, origin, civil status, household composition, previous benefit receipt, education, and work in the previous two years. These control variables served to test for balance from our randomisation procedure and were included in the regressions to reduce the variance of the estimator of the treatment effect [82]. We also used some of these variables to specify the relevant subgroups for our heterogeneity analysis. Finally, the participants were asked to express their satisfaction with the meeting; a question of general interest to the Social Services office.

We had an expert check the survey beforehand for functional illiteracy to maximise accessibility to the survey. The research was approved by the Ethics Committee of Maastricht University (ERCPN 176 05_02_ 2017). The survey took about 15–30 minutes.

84.5 per cent of the participants who agreed to participate in the study during the meeting also returned the survey. Our final sample consists of 174 individuals (104 in the treatment group and 70 in the control group) in 15 clusters. Table 1 provides a summary of socio-economic characteristics of our participants.

**Table 1:** Baseline summary statistics and test of balance for covariates.

	Mean	Test of randomisation balance		*N*
		Coefficient	*p*-value	
Age	38.35	21.38	0.064	174
Male	0.49	-0.38	0.467	86
Foreign background	0.28	0.31	0.501	48
Education				
No/special	0.06	0.06	0.805	11
Basic	0.10	0.13	0.698	18
Lower secondary	0.37	0.25	0.622	64
Higher secondary	0.29	-0.75	0.108	51
Tertiary	0.17	0.31	0.418	30
Household composition				
Single	0.47	0.63	0.215	81
Partner	0.09	0.00	1.000	15
Single parent	0.15	-0.75	0.033	26
Family	0.04	0.00	1.000	6
Other	0.24	0.13	0.761	46
Civil status				
Never married	0.68	-0.31	0.481	118
Married	0.11	0.00	1.000	19
Divorced or widowed	0.21	0.31	0.449	36
PW benefit in previous 2 years	0.27	-0.60	0.197	47
Paid work in previous 2 years	0.20	0.25	0.545	34
Quarter of intake meeting				
4/2017	0.39	0.31	0.527	68
1/2018	0.36	-0.75	0.057	63
2/2018	0.20	0.19	0.301	34
3/2018	0.04	0.25	0.614	6
4/2018	0.02	0.00	1.000	3

*Source*: Author’s own calculations.

*Notes*: All covariates are reported. The second column reports the sample mean. The third and fourth column report the coefficients and *p*-values from ordinary least squares regressions of each covariate on assignment to treatment, controlling for caseworker fixed effects. *N* = 174.

The average age is 38. The sample is evenly split between male and female participants. Three out of ten participants have a foreign background. The majority has completed lower or higher secondary education. Typically, individuals are single or single parents, or live with their parents or other relatives. The large majority has never been married. Roughly one fourth of applicants has had social assistance benefits in the last two years. Only one fifth had paid work in the last two years. The majority of participants applied for social assistance in the final quarter of 2017 or first quarter of 2018, consistent with the improved Dutch labour market situation after this period.

Table 1 also reports the results of a test of balance. Each covariate was regressed on a treatment assignment variable and caseworker fixed effects. With the exception of age, being a single parent, and application in the first quarter of 2018, none of the coefficients is significant at the 10-per cent level. The result for age is driven by the fact that there had been an unequal number of caseworkers in the youth team. With three of those caseworkers in the control and only two in the treatment group, the average age of applicants is higher in the treatment group.

### Analytical strategy

Our analyses are based on the intention-to-treat estimates of the effect of self-affirmation. Take-up rates are, however, close to 100 percent as the self-affirmation exercise was implemented in 100 out of the 104 meetings in the treatment group. The identification strategy takes into account the clustered random assignment. Failure to do so would lead to inflated Type-I error rates for the significance tests of regression coefficients, as the assumption of identically and independently distributed residuals would be ignored and standard errors underestimated [77, 80]. All socio-economic variables are included as control variables in the regression models.

The basic model is an ordinary-least-squares (OLS) regression with adjusted standard errors to reflect independence of observations across, but not necessarily within caseworkers. In order to appropriately model the small number of clusters and to understand the sensitivity of results to different methods, we use two additional models that perform well with few clusters [80]: multilevel and fixed effects models. In the multilevel models, we implement the Kenward-Roger adjustment that is recommended when sample size is small, the covariance structure is complicated and the data is unbalanced [91, 92] to avoid the inflation of Type-I errors. In the fixed effects models, we include indicator variables for each cluster to account for the nested nature of the data and recover the treatment effect by linear contrasts of the cluster affiliation variable coefficients. It is not possible to directly estimate the effects of Level-2 predictors, including the treatment effect, because of perfect multicollinearity of these predictors and the cluster affiliation predictors. Since the software does not accommodate the fact that there needs to be a distinction between variance at Level-1 and at Level-2, we multiply the standard errors of the output with the square root of the unconditional design effect (see [80] for more details). We report the results for these two models in the supplementary materials.

### Multiple hypotheses testing

We use data from a single experiment to test several null hypothesis arising from multiple outcomes of interest and subgroup analyses. Not correcting for multiple inference increases the likelihood of falsely rejecting the null hypothesis [93]. We use the Benjamini and Hochberg procedure to control the FDR. This means, in contrast to the family wise error rate (FWER) that reduces the probability of making any Type-I error but at the cost of greater power, we are willing to accept some Type-I errors in exchange for greater power [94]. Whereas the FWER is the probability that the number of false rejections is larger than 0, the FDR is the expected share of all rejections that are Type-I errors. As a result, *p*-value adjustments may be less strict when controlling the FDR at a given level, which in turn results in greater power [94]. This seems appropriate in the context of an exploratory study like ours that tests a newly designed intervention in a setting where self-affirmation has never been tested before.

The Benjamini-Hochberg procedure first puts all *p*-values in ascending order and ranks them from 1 to *m*, starting with the smallest *p*-value. For each individual *p*-value at rank *i*, the Benjamini-Hochberg critical value is calculated as (i/m)*Q, whereby *Q* indicates the chosen FDR. In the final step, the original *p*-values are compared to the critical values. The highest *p*-value that is smaller than the critical value, as well as all *p*-values that are smaller than this value, are considered significant. The analyses are carried out with Stata, using the *qqvalue* package [95]. In the supplementary materials we also report the results of the Romano-Wolf correction [96, 97], using the *rwolf* package in Stata.

## Results and discussion

**Fig 2:**
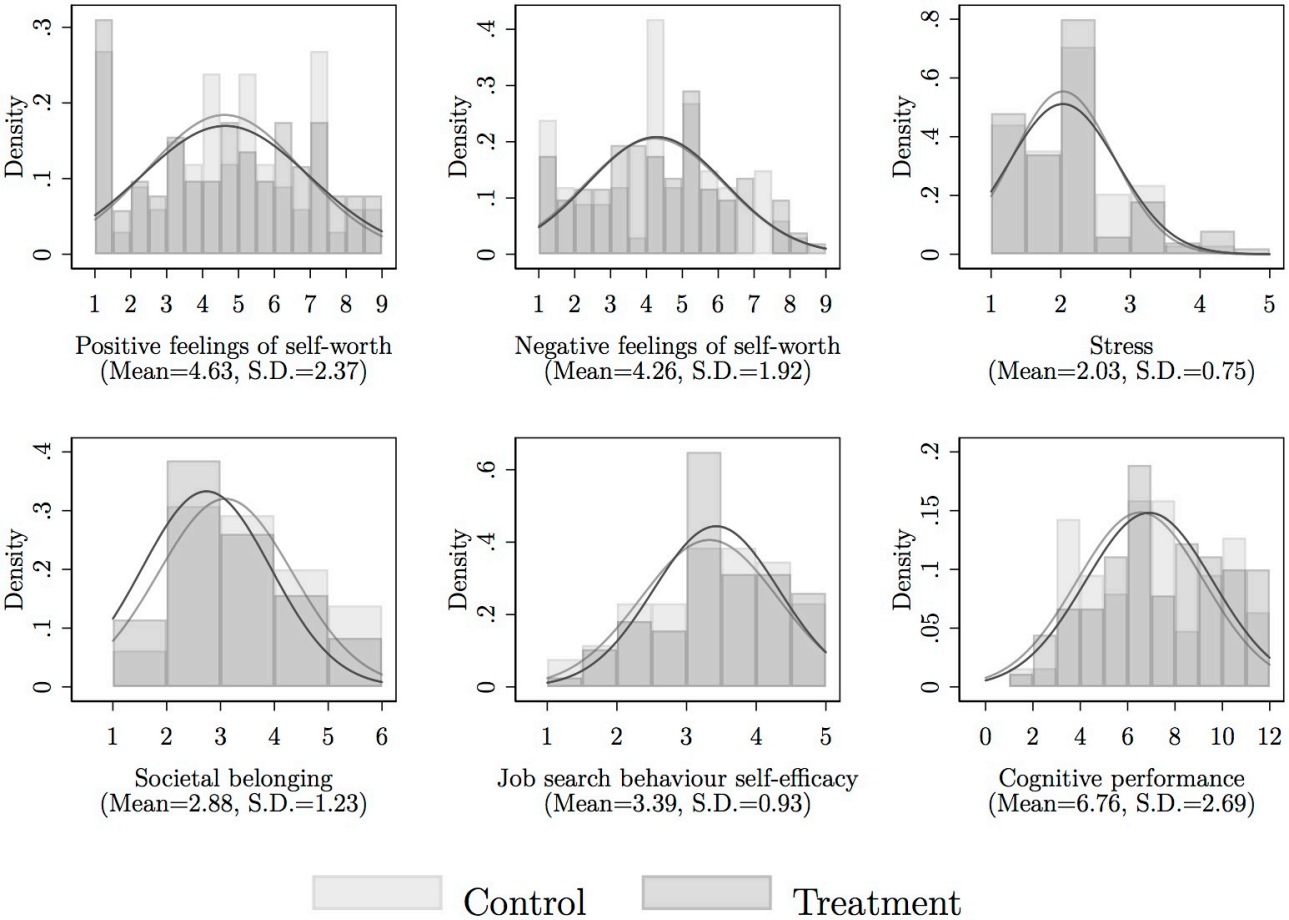
Histograms and kernel density of outcome variables, by treatment and control group. *Source*: Author’s own calculations.

Fig 2 presents non-parametric estimates for all outcome variables. Job search behaviour self-efficacy and cognitive performance are higher in the treatment group, whereas societal belonging appears to be higher in the control group. The difference is however only significant for feelings of societal belonging (*t*(134) = 1.789, *p* = 0.076).

**Fig 3:**
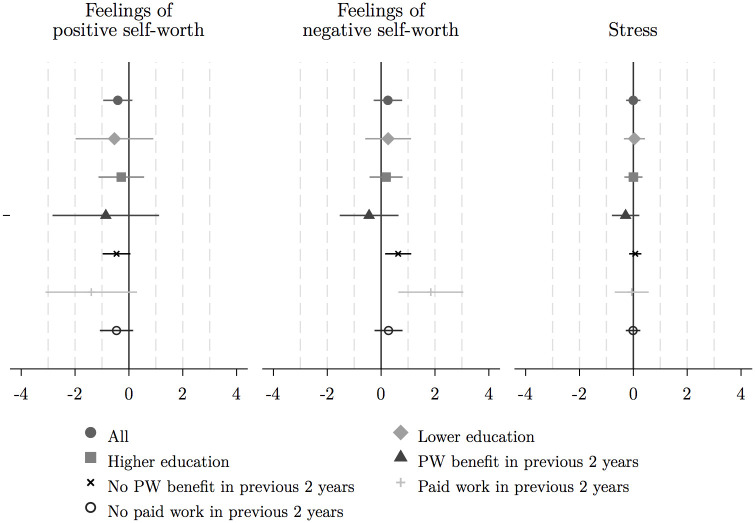
OLS estimation results, full sample and by subgroups: Positive and negative feelings of self-worth and stress. *Source*: Author’s own calculations. The markers indicate the point estimates and the extending lines the 90-per cent confidence intervals. In all models, control variables are included. The graph is created in Stata with the *coefplot* command developed by Jann [98].

**Fig 4:**
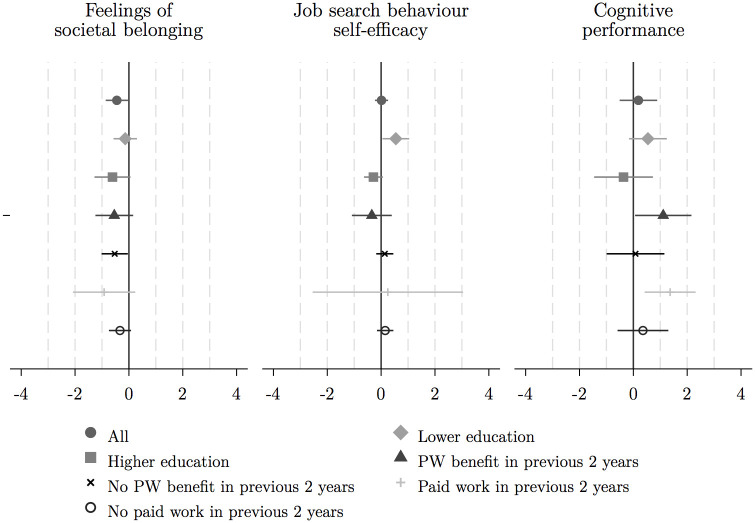
OLS estimation results, full sample and by subgroups: Feelings of societal belonging, job search behaviour self-efficacy and cognitive performance. *Source*: See Fig 3.

We now turn to our multivariate regression analysis. We find little support for an average effect of the affirmation exercise, except for a moderate negative effect on societal belonging. We therefore proceed with the subgroup analysis as average results may mask heterogeneous effects (see Figs 3 and 4 showing the OLS estimation results).

Indeed, the subgroup analysis reveals that the self-affirmation exercise increased negative feelings of self-worth among individuals who had not received social assistance in the past two years and for those who had paid work in that period. Also, the negative effect on feelings of societal belonging in the full sample seems to be driven by the group of individuals who had not received a social assistance benefit in the previous two years. Negative feelings for this group increase by approximately 0.4 standard deviations. We find positive impacts on cognitive performance for two groups: a 0.4–0.6 standard deviations increase for individuals that received benefits and for those who had paid work in the previous two years. Finally, self-affirmation boosted job search behaviour self-efficacy among individuals with lower levels of education (lower secondary and below) by approximately half a standard deviation (Fig 4), bringing them on par with average levels among individuals with higher levels of education.

The results are consistent across different specifications (see S1–S4 Figs). While point estimates are typically the same order of magnitude, the level of uncertainty differs across methods. Yet, with multiple outcomes and subgroups, it is pertinent to correct for multiple inference. Table 2 shows the adjusted *p*-values based on the Benjamini-Hochberg procedures for the whole sample for illustration. None of the results in the full sample remain significant, but results hold up to controlling the FDR (but not the FWER correction (see S2 Table)) for individuals that had paid work in the past two years.

**Table 2:** OLS estimation results, full sample and by paid/no paid work in previous two years: Multiple inference adjustments using Benjamini-Hochberg procedure.

	Effect	Naive *p*-value	FDR q-value: Benjamini-Hochberg
*Full sample*			
Positive feelings of self-worth	-0.412	0.202	0.606
Negative feelings of self-worth	0.252	0.415	0.830
Stress	-0.004	0.981	0.981
Societal belonging	-0.448	0.077	0.462
Job search behaviour self-efficacy	0.015	0.917	0.981
Cognitive performance	0.188	0.642	0.963
*Paid work in previous two years*			
Positive feelings of self-worth	-1.396	0.165	0.267
Negative feelings of self-worth	1.846	0.022	0.084
Stress	-0.061	0.863	0.871
Societal belonging	-0.920	0.178	0.267
Job search behaviour self-efficacy	0.249	0.871	0.871
Cognitive performance	1.366	0.028	0.084
*No paid work in previous two years*			
Positive feelings of self-worth	-0.460	0.213	0.594
Negative feelings of self-worth	0.275	0.368	0.594
Stress	-0.011	0.943	0.943
Societal belonging	-0.331	0.178	0.594
Job search behaviour self-efficacy	0.151	0.396	0.594
Cognitive performance	0.356	0.516	0.619

*Source*: Author’s own calculations.

*Notes*: Control variables are included in all models.

Overall we find little evidence that self-affirmation has beneficial effects for our target population. If anything, the results suggest self-affirmation had a negative impact on certain groups. For individuals with better labour market perspectives—indicated by being in paid work in the previous two years—for example, their personal past might influence their present experience of relative deprivation [99] and the affirmation might unintentionally be task-relevant. This is consistent with informal feedback on chosen self-affirmation topics that we received from caseworkers, stating that a substantial group of participants talked about a work-related experience. Although the protocol explicitly stipulated that participants should choose a domain unrelated to work, the mentioning of “work” may have acted as a negative prime for some people, reminding them of their (recent) past as employees and thereby negatively affecting their self-worth.

The observed increase in cognitive performance for this group is possibly induced by an urge to prove and distinguish themselves from other applicants. Rather than restoring a “damaged identity”, the self-affirmation exercise might make it loom larger and emphasises the salience of their new identity as social service applicants—a group that is threatened by negative stereotypes and stigma in society [43]. We know from studies on social identity that status differences between groups may indeed influence efforts in response to salience of identity, threats to the hierarchy status quo and distinctiveness [100–102]. Or, they have not yet come to terms with this new identity and still perceive themselves as a representative member of their old and arguably higher-valued “in-group”, that is, being part of the the labour force. If this type of self-categorisation comes under threat, we may then also see increasing efforts when individuals feel at risk of being classified as a member of a lower-status or less valued group, especially when group boundaries are highly permeable [100, 103, 104].

## Conclusion

Self-affirmation has been promoted as a low-cost and easy-to-implement effective tool for restoring personal integrity situations where this is challenged by stigma and stereotype threats. We provide the first evidence on the role of self-affirmation in the realm of welfare assistance. We developed a new verbal self-affirmation intervention and tested it among a sample of 174 social assistance applicants in Maastricht, the Netherlands. Overall, the self-affirmation has no average impact on individuals’ feelings of self-worth, stress, job search behaviour, self-efficacy or cognitive performance, but negatively affects feelings of societal belonging in our full sample.

Subgroup analyses suggest heterogenous treatment effects, depending on individual’s personal past and education. However, only two results hold up to multiple hypothesis testing. These results suggest that self-affirmation in this context may even be harmful, testified by increased negative feelings of self-worth among people considered closer to the labour market. Our results are sobering and at odds with the overwhelmingly positive findings of self-affirmation interventions in other studies. This cautions against using such an exercise for specific groups in the context under study. An interesting question for future studies could be to experimentally investigate whether threats to social identity and self-categorisation mechanisms may indeed be at work, and whether using another type or procedure for self-affirmation may perhaps make it easier for people to self-affirm in a positive way and generate desirable impacts on beliefs, attitudes and behaviour. This is left for future work.

## Supporting information

S1 Table Intracluster correlation coefficient. (PDF)

S2 Table OLS estimation results, full sample and by paid/no paid work in previous two years: Multiple inference adjustments using Romano-Wolf correction. (PDF)

S1 Fig Multilevel model estimation results, full sample and by subgroups: Positive and negative feelings of self-worth and stress. *Source*: See Fig 3.
(TIF)

S2 Fig Multilevel model estimation results, full sample and by subgroups: Feelings of societal belonging, job search behaviour self-efficacy and cognitive performance. *Source*: See Fig 3.
(TIF)

S3 Fig Fixed effects model estimation results, full sample and by subgroups: Positive and negative feelings of self-worth and stress. *Source*: See Fig 3.
(TIF)

S4 Fig Fixed effects model estimation results, full sample and by subgroups: Feelings of societal belonging, job search behaviour self-efficacy and cognitive performance. *Source*: See Fig 3.
(TIF)

## References

[1] Spencer SJ, Fein S, Lomore CD. Maintaining one’s self-image vis-à-vis others: The role of self-affirmation in the social evaluation of the self. Motivation and Emotion. 2001;25(1):41–65. doi: 10.1023/A:1010659805978

[2] Martens A, Johns M, Greenberg J, Schimel J. Combating stereotype threat: The effect of self-affirmation on women’s intellectual performance. Journal of Experimental Social Psychology. 2006;42(2):236–243. doi: 10.1016/j.jesp.2005.04.010

[3] Leyens JP, Désert M, Croizet JC, Darcis C. Stereotype threat: Are lower status and history of stigmatization preconditions of stereotype threat? Personality and Social Psychology Bulletin. 2000;26(10):1189–1199. doi: 10.1177/0146167200262002

[4] Crocker J, Niiya Y, Mischkowski D. Why does writing about important values reduce defensiveness? Self-affirmation and the role of positive other-directed feelings. Psychological Science. 2008;19(7):740–747. doi: 10.1111/j.1467-9280.2008.02150.x 18727791

[5] Major B, O’brien LT. The social psychology of stigma. Annual Review of Psychology. 2005;56:393–421. doi: 10.1146/annurev.psych.56.091103.070137 15709941

[6] Horan PM, Austin PL. The social bases of welfare stigma. Social Problems. 1974;21(5):648–657. doi: 10.2307/799640

[7] Goodban N. The psychological impact of being on welfare. Social Service Review. 1985;59(3):403–422. doi: 10.1086/644308

[8] Rogers-Dillon R. The dynamics of welfare stigma. Qualitative Sociology. 1995;18(4):439. doi: 10.1007/BF02404490

[9] Lister R. Poverty. Cambridge, United Kingdom: Polity; 2004.

[10] Lister R. ‘To count for nothing’: Poverty beyond the statistics. Journal of the British Academy. 2015;3:139–165.

[11] Wright S. Conceptualising the active welfare subject: Welfare reform in discourse, policy and lived experience. Policy & Politics. 2016;44(2):235–252. doi: 10.1332/030557314X13904856745154

[12] Garthwaite K. Hunger pains. Life inside foodbank Britain. Bristol, United Kingdom: Policy Press; 2016.

[13] Gubrium E, Lødemel I. ‘Not good enough’: Social assistance and shaming in Norway. In: Gubrium E, Pellissery S, Lødemel I, editors. The shame of it: Global perspectives on anti-poverty policies. Bristol, United Kingdom: Policy Press; 2014. p. 85–110.

[14] Kampen T, Elshout J, Tonkens E. The fragility of self-respect: Emotional labour of workfare volunteering. Social Policy and Society. 2013;12(3):427–438. doi: 10.1017/S1474746413000067

[15] Ohls C. A qualitative study exploring matters of ill-being and well-being in Norwegian activation policy. Social Policy and Society. 2017;16(4):593–606. doi: 10.1017/S1474746416000397

[16] Patrick R. For whose benefit? The everyday realities of welfare reform. Bristol, United Kingdom: Policy Press; 2017.

[17] Patrick R. Living with and responding to the ‘scrounger’ narrative in the UK: Exploring everyday strategies of acceptance, resistance and deflection. Journal of Poverty and Social Justice. 2016;24(3):245–259. doi: 10.1332/175982716X14721954314887

[18] Baumberg B. The stigma of claiming benefits: A quantitative study. Journal of Social Policy. 2016;45(2):181–199. doi: 10.1017/S0047279415000525

[19] Bertrand M, Luttmer EF, Mullainathan S. Network effects and welfare cultures. The Quarterly Journal of Economics. 2000;115(3):1019–1055. doi: 10.1162/003355300554971

[20] Lee WS, Oguzoglu U. Income support and stigma effects for young Australians. Australian Economic Review. 2007;40(4):369–384. doi: 10.1111/j.1467-8462.2007.00477.x

[21] Handler JF, Hollingsworth EJ. Stigma, privacy, and other attitudes of welfare recipients. Stanford Law Review. 1969;22(1):1–19. doi: 10.2307/1227402

[22] Besley T, Coate S. Understanding welfare stigma: Taxpayer resentment and statistical discrimination. Journal of Public Economics. 1992;48(2):165–183. doi: 10.1016/0047-2727(92)90025-B

[23] Fiske ST, Cuddy AJ, Glick P, Xu J. A model of (often mixed) stereotype content: Competence and warmth respectively follow from perceived status and competition. Journal of Personality and Social Psychology. 2002;82(6):878–902. doi: 10.1037/0022-3514.82.6.878 12051578

[24] Cuddy AJ, Fiske ST, Glick P. The BIAS map: Behaviors from intergroup affect and stereotypes. Journal of Personality and Social Psychology. 2007;92(4):631–648. doi: 10.1037/0022-3514.92.4.631 17469949

[25] Contini D, Richiardi MG. Reconsidering the effect of welfare stigma on unemployment. Journal of Economic Behavior & Organization. 2012;84(1):229–244. doi: 10.1016/j.jebo.2012.02.010

[26] Moffitt R, et al. An economic model of welfare stigma. American Economic Review. 1983;73(5):1023–1035.

[27] Currie JM, Grogger J. Explaining recent declines in food stamp program participation. Brookings-Wharton Papers on Urban Affairs. 2001; p. 203–244.

[28] Currie JM. The invisible safety net: Protecting the nation’s poor children and families. Princeton, NJ: Princeton University Press; 2006.

[29] Bane MJ, Ellwood DT. Welfare realities: From rhetoric to reform. Cambridge, MA: Harvard University Press; 1996.

[30] Gennetian LA, Shafir E. The persistence of poverty in the context of financial instability: A Behavioral Perspective. Journal of Public Policy Analysis and Management. 2015;34(4):904–936. doi: 10.1002/pam.21854

[31] Sheehy-Skeffington J, Rea J. How poverty affects people’s decision-making processes. Joseph Rowntree Foundaction; 2017.

[32] Shafir E. Decisions in poverty contexts. Current Opinion in Psychology. 2017;18:131–136. doi: 10.1016/j.copsyc.2017.08.026 28923664

[33] Haushofer J, Fehr E. On the psychology of poverty. Science. 2014;344(6186):862–867. doi: 10.1126/science.1232491 24855262

[34] Kunze L, Suppa N. Bowling alone or bowling at all? The effect of unemployment on social participation. Journal of Economic Behavior & Organization. 2017;133:213–235. doi: 10.1016/j.jebo.2016.11.012

[35] Steele CM. The psychology of self-affirmation: Sustaining the integrity of the self. Advances in Experimental Social Psychology. 1988;21:261–302.

[36] Cohen GL, Sherman DK. The psychology of change: Self-affirmation and social psychological intervention. Annual Review of Psychology. 2014;65:333–371. doi: 10.1146/annurev-psych-010213-115137 24405362

[37] Hall CC, Zhao J, Shafir E. Self-affirmation among the poor: Cognitive and behavioral implications. Psychological Science. 2014;25(2):619–625. doi: 10.1177/0956797613510949 24357617

[38] Stuber J, Schlesinger M. Sources of stigma for means-tested government programs. Social Science & Medicine. 2006;63(4):933–945. doi: 10.1016/j.socscimed.2006.01.012 16542766

[39] Sherman DK. Self-affirmation: Understanding the effects. Social and Personality Psychology Compass. 2013;7(11):834–845. doi: 10.1111/spc3.12072

[40] Sherman DK, Cohen GL. The psychology of self-defense: Self-affirmation theory. Advances in Experimental Social Psychology. 2006;38:183–242.

[41] Schmader T, Johns M, Forbes C. An integrated process model of stereotype threat effects on performance. Psychological Review. 2008;115(2):336–356. doi: 10.1037/0033-295X.115.2.336 18426293 PMC2570773

[42] McQueen A, Klein WM. Experimental manipulations of self-affirmation: A systematic review. Self and Identity. 2006;5(4):289–354. doi: 10.1080/15298860600805325

[43] Critcher CR, Dunning D. Self-affirmations provide a broader perspective on self-threat. Personality and Social Psychology Bulletin. 2015;41(1):3–18. doi: 10.1177/0146167214554956 25319717

[44] Schmeichel BJ, Vohs K. Self-affirmation and self-control: Affirming core values counteracts ego depletion. Journal of Personality and Social Psychology. 2009;96(4):770–782. doi: 10.1037/a0014635 19309201

[45] Liu TJ, Steele CM. Attributional analysis as self-affirmation. Journal of Personality and Social Psychology. 1986;51(3):531. doi: 10.1037/0022-3514.51.3.531

[46] Klein WM, Harris PR. Self-affirmation enhances attentional bias toward threatening components of a persuasive message. Psychological Science. 2009;20(12):1463–1467. doi: 10.1111/j.1467-9280.2009.02467.x 19883488

[47] Reed MB, Aspinwall LG. Self-affirmation reduces biased processing of health-risk information. Motivation and Emotion. 1998;22(2):99–132. doi: 10.1023/A:1021463221281

[48] Cohen GL, Garcia J, Purdie-Vaughns V, Apfel N, Brzustoski P. Recursive processes in self-affirmation: Intervening to close the minority achievement gap. Science. 2009;324(5925):400–403. doi: 10.1126/science.1170769 19372432

[49] Miyake A, Kost-Smith LE, Finkelstein ND, Pollock SJ, Cohen GL, Ito TA. Reducing the gender achievement gap in college science: A classroom study of values affirmation. Science. 2010;330(6008):1234–1237. doi: 10.1126/science.1195996 21109670

[50] Sherman DK, Hartson KA, Binning KR, Purdie-Vaughns V, Garcia J, Taborsky-Barba S, et al. Deflecting the trajectory and changing the narrative: How self-affirmation affects academic performance and motivation under identity threat. Journal of Personality and Social Psychology. 2013;104(4):591–618. doi: 10.1037/a0031495 23397969

[51] Wiesenfeld BM, Brockner J, Petzall B, Wolf R, Bailey J. Stress and coping among layoff survivors: A self-affirmation analysis. Anxiety, Stress and Coping. 2001;14(1):15–34. doi: 10.1080/10615800108248346

[52] Jiang L. Job insecurity and creativity: The buffering effect of self-affirmation and work-affirmation. Journal of Applied Social Psychology. 2018;48(7):388–397. doi: 10.1111/jasp.12519

[53] Moeini-Jazani M, Albalooshi S, Seljeseth IM. Self-affirmation reduces delay discounting of the financially deprived. Frontiers in Psychology. 2019;10:1729. doi: 10.3389/fpsyg.2019.01729 31417458 PMC6682614

[54] Banker S, Bhanot SP, Deshpande A. Poverty identity and preference for challenge: Evidence from the US and India. Journal of Economic Psychology. 2020;76:102214. doi: 10.1016/j.joep.2019.102214

[55] Creswell JD, Welch WT, Taylor SE, Sherman DK, Gruenewald TL, Mann T. Affirmation of personal values buffers neuroendocrine and psychological stress responses. Psychological Science. 2005;16(11):846–851. doi: 10.1111/j.1467-9280.2005.01624.x 16262767

[56] Harris PR, Mayle K, Mabbott L, Napper L. Self-affirmation reduces smokers’ defensiveness to graphic on-pack cigarette warning labels. Health Psychology. 2007;26(4):437–446. doi: 10.1037/0278-6133.26.4.437 17605563

[57] Klein WM, Harris PR, Ferrer RA, Zajac LE. Feelings of vulnerability in response to threatening messages: Effects of self-affirmation. Journal of Experimental Social Psychology. 2011;47(6):1237–1242. doi: 10.1016/j.jesp.2011.05.005

[58] Reavis RD, Ebbs JB, Onunkwo AK, Sage LM. A self-affirmation exercise does not improve intentions to vaccinate among parents with negative vaccine attitudes (and may decrease intentions to vaccinate). PLoS One. 2017;12(7):e0181368. doi: 10.1371/journal.pone.0181368 28704520 PMC5509329

[59] Dee TS. Social identity and achievement gaps: Evidence from an affirmation intervention. Journal of Research on Educational Effectiveness. 2015;8(2):149–168. doi: 10.1080/19345747.2014.906009

[60] Protzko J, Aronson J. Context moderates affirmation effects on the ethnic achievement gap. Social Psychological and Personality Science. 2016;7(6):500–507. doi: 10.1177/1948550616646426

[61] Hanselman P, Rozek CS, Grigg J, Borman GD. New evidence on self-affirmation effects and theorized sources of heterogeneity from large-scale replications. Journal of Educational Psychology. 2017;109(3):405. doi: 10.1037/edu0000141 28450753 PMC5403146

[62] Sherman DK, Cohen GL. Accepting threatening information: Self-affirmation and the reduction of defensive biases. Current Directions in Psychological Science. 2002;11(4):119–123. doi: 10.1111/1467-8721.00182

[63] Sherman DK, Bunyan DP, Creswell JD, Jaremka LM. Psychological vulnerability and stress: The effects of self-affirmation on sympathetic nervous system responses to naturalistic stressors. Health Psychology. 2009;28(5):554–562. doi: 10.1037/a0014663 19751081

[64] Creswell JD, Lam S, Stanton AL, Taylor SE, Bower JE, Sherman DK. Does self-affirmation, cognitive processing, or discovery of meaning explain cancer-related health benefits of expressive writing? Personality and Social Psychology Bulletin. 2007;33(2):238–250. doi: 10.1177/0146167206294412 17259584

[65] Cook JE, Purdie-Vaughns V, Garcia J, Cohen GL. Chronic threat and contingent belonging: Protective benefits of values affirmation on identity development. Journal of Personality and Social Psychology. 2012;102(3):479–496. doi: 10.1037/a0026312 22082058

[66] Bandura A. Self-efficacy. In: Ramachaudran VS, editor. Encyclopedia of Human Behavior. vol. 4. New York, NY: Academic Press; 1994. p. 71–81.

[67] Liu S, Huang JL, Wang M. Effectiveness of job search interventions: A meta-analytic review. Psychological Bulletin. 2014;140(4):1009–1041. doi: 10.1037/a0035923 24588365

[68] Epton T, Harris PR. Self-affirmation promotes health behavior change. Health Psychology. 2008;27(6):746–752. doi: 10.1037/0278-6133.27.6.746 19025270

[69] Zhao X, Nan X. Influence of self-affirmation on responses to gain-versus loss-framed antismoking messages. Human Communication Research. 2010;36(4):493–511. doi: 10.1111/j.1468-2958.2010.01385.x

[70] Saks AM, Zikic J, Koen J. Job search self-efficacy: Reconceptualizing the construct and its measurement. Journal of Vocational Behavior. 2015;86:104–114. doi: 10.1016/j.jvb.2014.11.007

[71] Walton GM, Spencer SJ. Latent ability grades and test scores systematically underestimate the intellectual ability of negatively stereotyped students. Psychological Science. 2009;20(9):1132–1139. doi: 10.1111/j.1467-9280.2009.02417.x 19656335

[72] Creswell JD, Dutcher JM, Klein WM, Harris PR, Levine JM. Self-affirmation improves problem-solving under stress. PLoS One. 2013;8(5):1–7. doi: 10.1371/journal.pone.0062593 23658751 PMC3641050

[73] Cohen GL, Garcia J, Apfel N, Master A. Reducing the racial achievement gap: A social-psychological intervention. Science. 2006;313(5791):1307–1310. doi: 10.1126/science.1128317 16946074

[74] Ehret PJ, LaBrie JW, Santerre C, Sherman DK. Self-affirmation and motivational interviewing: Integrating perspectives to reduce resistance and increase efficacy of alcohol interventions. Health Psychology Review. 2015;9(1):83–102. doi: 10.1080/17437199.2013.840953 25793492

[75] Centraal Bureau voor de Statistiek. StatLine; 2019.

[76] Donner A, Klar N. Pitfalls of and controversies in cluster randomization trials. American Journal of Public Health. 2004;94(3):416–422. doi: 10.2105/ajph.94.3.416 14998805 PMC1448267

[77] Hayes RJ, Moulton LH. Cluster randomised trials. Boca Raton, FL: Chapman &Hall/CRC Press; 2009.

[78] Wears RL. Advanced statistics: Statistical methods for analyzing cluster and cluster-randomized data. Academic Emergency Medicine. 2002;9(4):330–341. doi: 10.1197/aemj.9.4.330 11927463

[79] Torgerson DJ. Contamination in trials: is cluster randomisation the answer? British Medical Journal. 2001;322(7282):355–357. doi: 10.1136/bmj.322.7282.355 11159665 PMC1119583

[80] McNeish D, Stapleton LM. Modeling clustered data with very few clusters. Multivariate Behavioral Research. 2016;51(4):495–518. doi: 10.1080/00273171.2016.1167008 27269278

[81] Borm GF, Melis RJ, Teerenstra S, Peer PG. Pseudo cluster randomization: A treatment allocation method to minimize contamination and selection bias. Statistics in Medicine. 2005;24(23):3535–3547. doi: 10.1002/sim.2200 16007575

[82] Duflo E, Glennerster R, Kremer M. Using randomization in development economics research: A toolkit. In: Schultz T, Strauss J, editors. Handbook of development economics. vol. 4. Amsterdam, the Netherlands: North Holland; 2008. p. 3895–3962.

[83] Karlan D, Appel J. Failing in the field: What we can learn when field research goes wrong. Princeton, NJ: Princeton University Press; 2016.

[84] Malmberg-Heimonen I, Tøge AG. Effects of individualised follow-up on activation programme participants’ self-sufficiency: A cluster-randomised study. International Journal of Social Welfare. 2016;25(1):27–35. doi: 10.1111/ijsw.12179

[85] Behncke S, Frölich M, Lechner M. Targeting labour market programmes: Results from a randomized experiment. Swiss Journal of Economics and Statistics. 2009;145(3):221–268. doi: 10.1007/BF03399281

[86] Dong N, Maynard R. PowerUp!: A tool for calculating minimum detectable effect sizes and minimum required sample sizes for experimental and quasi-experimental design studies. Journal of Research on Educational Effectiveness. 2013;6(1):24–67. doi: 10.1080/19345747.2012.673143

[87] Carril A. Dealing with misfits in random treatment assignment. The Stata Journal. 2017;17(3):652–667. doi: 10.1177/1536867X1701700307

[88] Mashek D, Cannaday LW, Tangney JP. Inclusion of community in self scale: A single-item pictorial measure of community connectedness. Journal of Community Psychology. 2007;35(2):257–275. doi: 10.1002/jcop.20146

[89] Gächter S, Starmer C, Tufano F. Measuring the closeness of relationships: A comprehensive evaluation of the’Inclusion of the Other in the Self’scale. PloS one. 2015;10(6). 10.1371/journal.pone.0129478 PMC4466912 26068873

[90] Bilker WB, Hansen JA, Brensinger CM, Richard J, Gur RE, Gur RC. Development of abbreviated nine-item forms of the Raven’s Standard Progressive Matrices Test. Assessment. 2012;19(3):354–369. doi: 10.1177/1073191112446655 22605785 PMC4410094

[91] Yang X. Small-sample inference for linear mixed-effects models; 2015. Presentation at 2015 Stata Conference.

[92] McNeish D, Stapleton LM. The effect of small sample size on two-level model estimates: A review and illustration. Educational Psychology Review. 2016;28(2):295–314. doi: 10.1007/s10648-014-9287-x

[93] List JA, Shaikh AM, Xu Y. Multiple hypothesis testing in experimental economics. Experimental Economics. 2019;22(4):773–793. doi: 10.1007/s10683-018-09597-5

[94] Anderson ML. Multiple inference and gender differences in the effects of early intervention: A reevaluation of the Abecedarian, Perry Preschool, and Early Training Projects. Journal of the American Statistical Association. 2008;103(484):1481–1495. doi: 10.1198/016214508000000841

[95] Newson RB. Frequentist q-values for multiple-test procedures. The Stata Journal. 2010;10(4):568–584. doi: 10.1177/1536867X1001000403

[96] Romano JP, Wolf M. Efficient computation of adjusted p-values for resampling-based stepdown multiple testing. Statistics & Probability Letters. 2016;113:38–40. doi: 10.1016/j.spl.2016.02.012

[97] Clarke D, Romano JP, Wolf M. The Romano-Wolf multiple-hypothesis correction in Stata. The Stata Journal. 2020;20(4):812–843. doi: 10.1177/1536867X20976314

[98] Jann B. Plotting regression coefficients and other estimates. The Stata Journal. 2014;14(4):708–737. doi: 10.1177/1536867X1401400402

[99] Crosby F. A model of egoistical relative deprivation. Psychological Review. 1976;83(2):85–113. doi: 10.1037/0033-295X.83.2.85

[100] Branscombe NR, Ellemers N, Spears R, Doosje B, et al. The context and content of social identity threat. Social identity: Context, commitment, content. 1999; p. 35–58.

[101] Scheepers D, Ellemers N. When the pressure is up: The assessment of social identity threat in low and high status groups. Journal of Experimental Social Psychology. 2005;41(2):192–200. doi: 10.1016/j.jesp.2004.06.002

[102] Haslam SA, Reicher S. Stressing the group: social identity and the unfolding dynamics of responses to stress. Journal of Applied Psychology. 2006;91(5):1037–1052. doi: 10.1037/0021-9010.91.5.1037 16953766

[103] Schmitt MT, Branscombe NR. The good, the bad, and the manly: Threats to one’s prototypicality and evaluations of fellow in-group members. Journal of Experimental Social Psychology. 2001;37(6):510–517. doi: 10.1006/jesp.2001.1476

[104] Pettit NC, Lount RB Jr. Looking down and ramping up: The impact of status differences on effort in intergroup contexts. Journal of Experimental Social Psychology. 2010;46(1):9–20. doi: 10.1016/j.jesp.2009.08.008

