# Supplementary material for: Sustaining the integrity of the threatened self: A cluster-randomised trial among social assistance applicants in the Netherlands
Source: PLoS One. 2021 Jun 3;16(6):e0252268. doi: 10.1371/journal.pone.0252268 (PMC8174741; doi:10.1371/journal.pone.0252268)
Supplement: S1 Table — (PDF) [file pone.0252268.s001.pdf]

S1 Table. Intracluster correlation coefficients

	All		Treatment		Control	
	ICC	S.E.	ICC	S.E.	ICC	S.E.
Positive feelings self-worth	0.000	0.036	0.035	0.059	0.000	0.074
Negative feelings self-worth	0.000	0.036	0.000	0.042	0.017	0.081
Stress	0.072	0.060	0.036	0.061	0.183	0.145
Societal belonging	0.050	0.054	0.109	0.010	0.000	0.072
Job search behaviour self-efficacy	0.046	0.062	0.116	0.110	0.000	0.098
Cognitive performance	0.028	0.049	0.000	0.048	0.147	0.138

Source: Author's own calculations.
Notes: The second to seventh column show the intracluster correlation coefficients (ICC) and the asymptotic standard errors (S.E.) for all outcome variables; for the full sample and the treatment and control groups separately.
