# Supplementary material for: Sustaining the integrity of the threatened self: A cluster-randomised trial among social assistance applicants in the Netherlands
Source: PLoS One. 2021 Jun 3;16(6):e0252268. doi: 10.1371/journal.pone.0252268 (PMC8174741; doi:10.1371/journal.pone.0252268)
Supplement: S2 Table — (PDF) [file pone.0252268.s002.pdf]

S6 Table. OLS estimation results, full sample and by paid/no paid work in previous two years: Multiple inference adjustments using Romano-Wolf correction

	Effect	Naive p-value	FWER p-value: Romano-Wolf
Full sample			
Positive feelings of self-worth	-0.412	0.202	0.702
Negative feelings of self-worth	0.252	0.415	0.893
Stress	-0.004	0.981	0.997
Societal belonging	-0.448	0.077	0.521
Job search behaviour self-efficacy	0.015	0.917	0.997
Cognitive performance	0.188	0.642	0.957
Paid work in previous two years			
Positive feelings of self-worth	-1.396	0.165	0.967
Negative feelings of self-worth	1.846	0.022	0.963
Stress	-0.061	0.863	0.983
Societal belonging	-0.920	0.178	0.967
Job search behaviour self-efficacy	0.249	0.871	0.983
Cognitive performance	1.366	0.028	0.963
No paid work in previous two years			
Positive feelings of self-worth	-0.460	0.213	0.723
Negative feelings of self-worth	0.275	0.368	0.827
Stress	-0.011	0.943	0.943
Societal belonging	-0.331	0.178	0.723
Job search behaviour self-efficacy	0.151	0.396	0.827
Cognitive performance	0.356	0.516	0.827

Source: Author's own calculations.
Notes: In all models, control variables are included. 1000 bootstrap replications performed.
