# Supplementary figures and images for: Sustaining the integrity of the threatened self: A cluster-randomised trial among social assistance applicants in the Netherlands
Source: PLoS One. 2021 Jun 3;16(6):e0252268. doi: 10.1371/journal.pone.0252268 (PMC8174741; doi:10.1371/journal.pone.0252268)

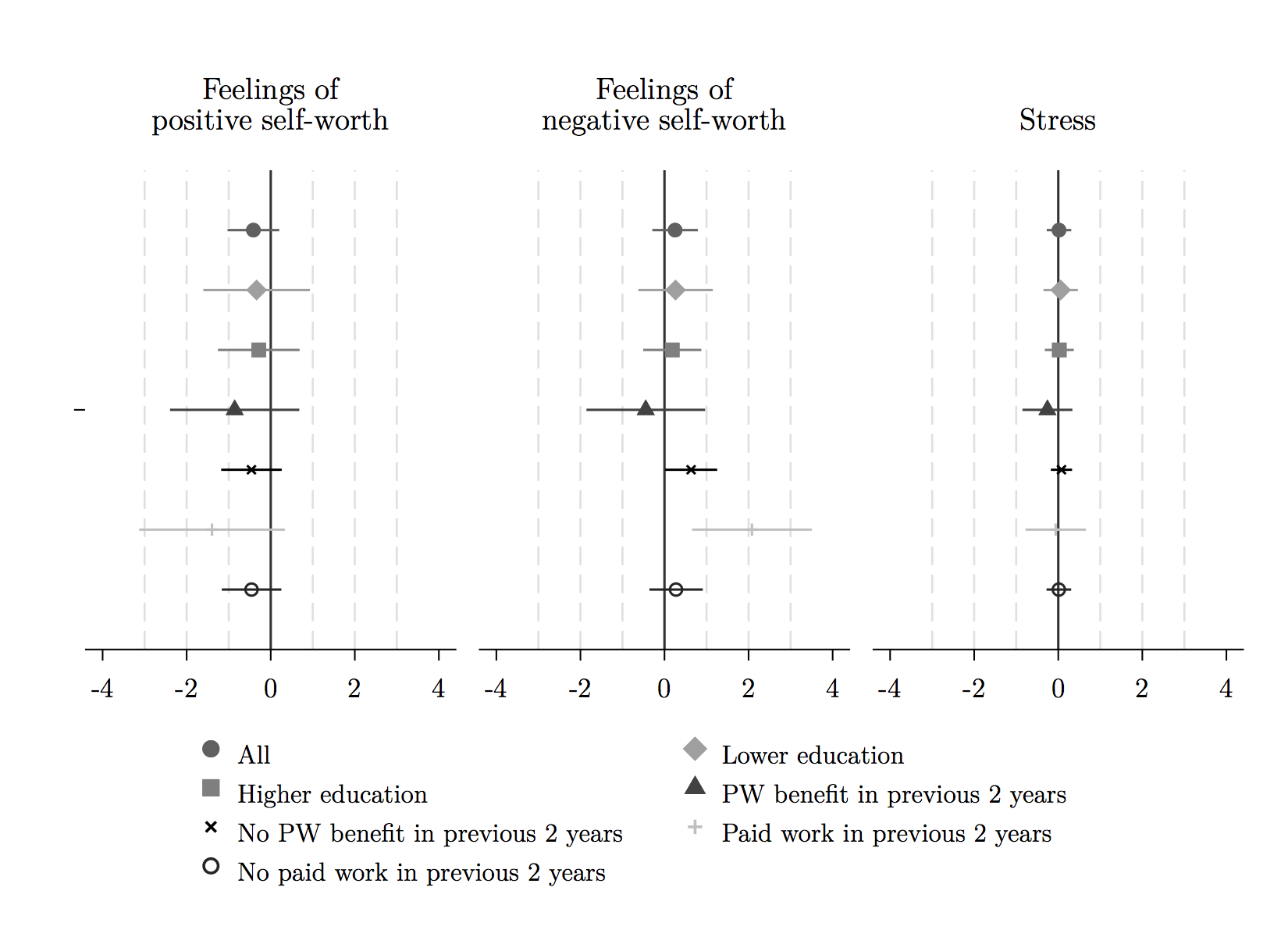

Supplement: S1 Fig — Source: See Fig 3. (TIF) [file pone.0252268.s003.tif]

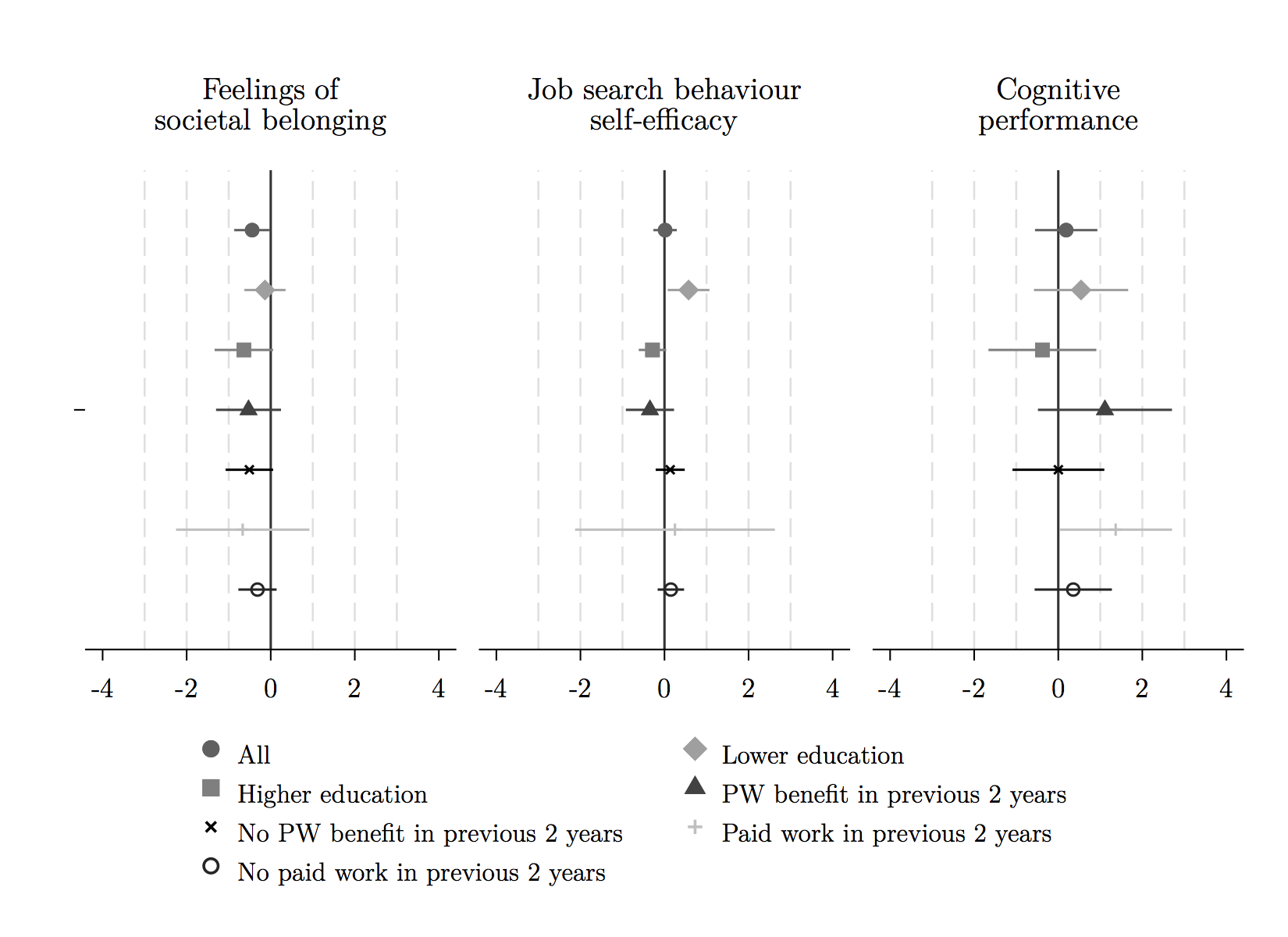

Supplement: S2 Fig — Source: See Fig 3. (TIF) [file pone.0252268.s004.tif]

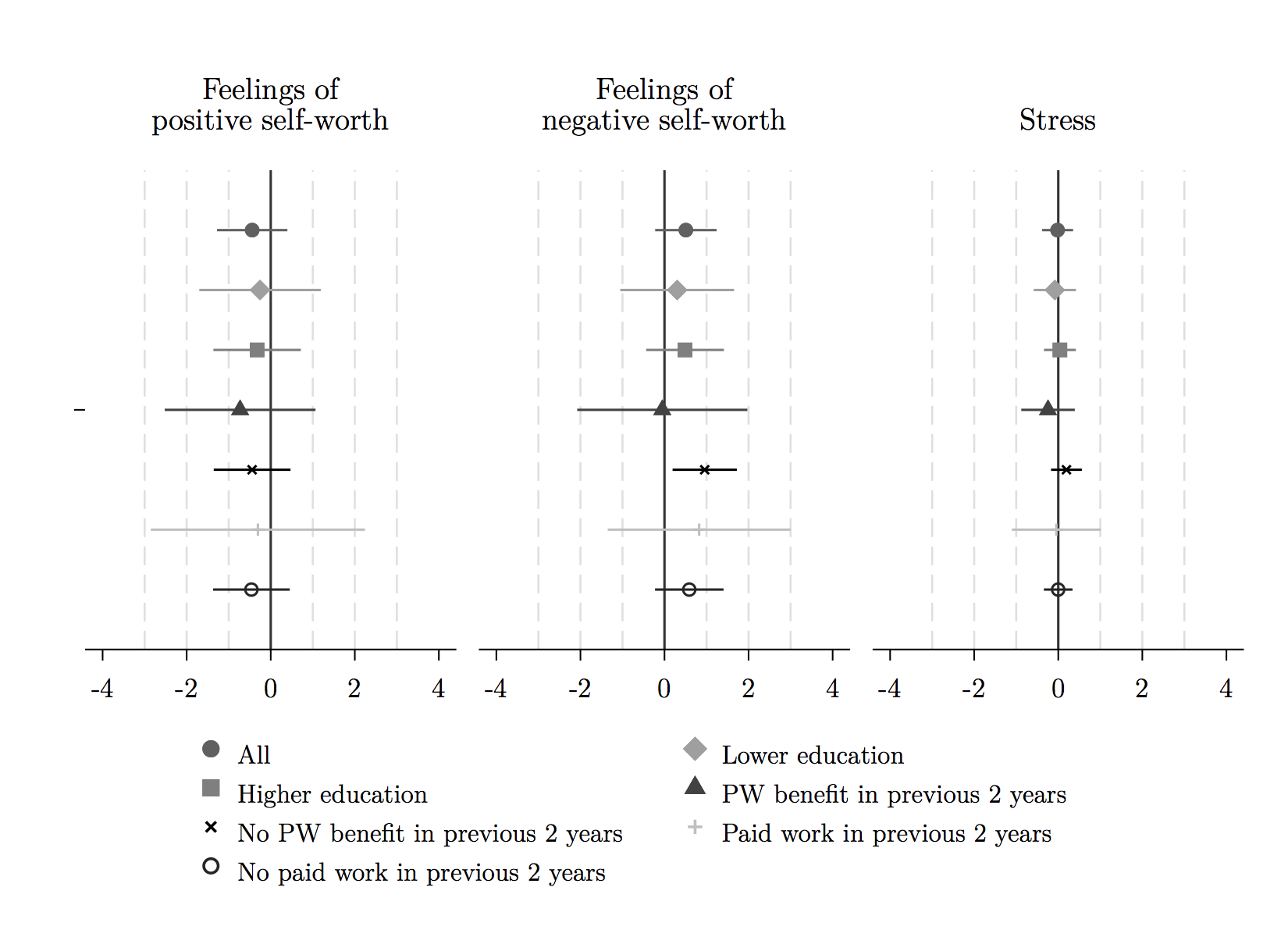

Supplement: S3 Fig — Source: See Fig 3. (TIF) [file pone.0252268.s005.tif]

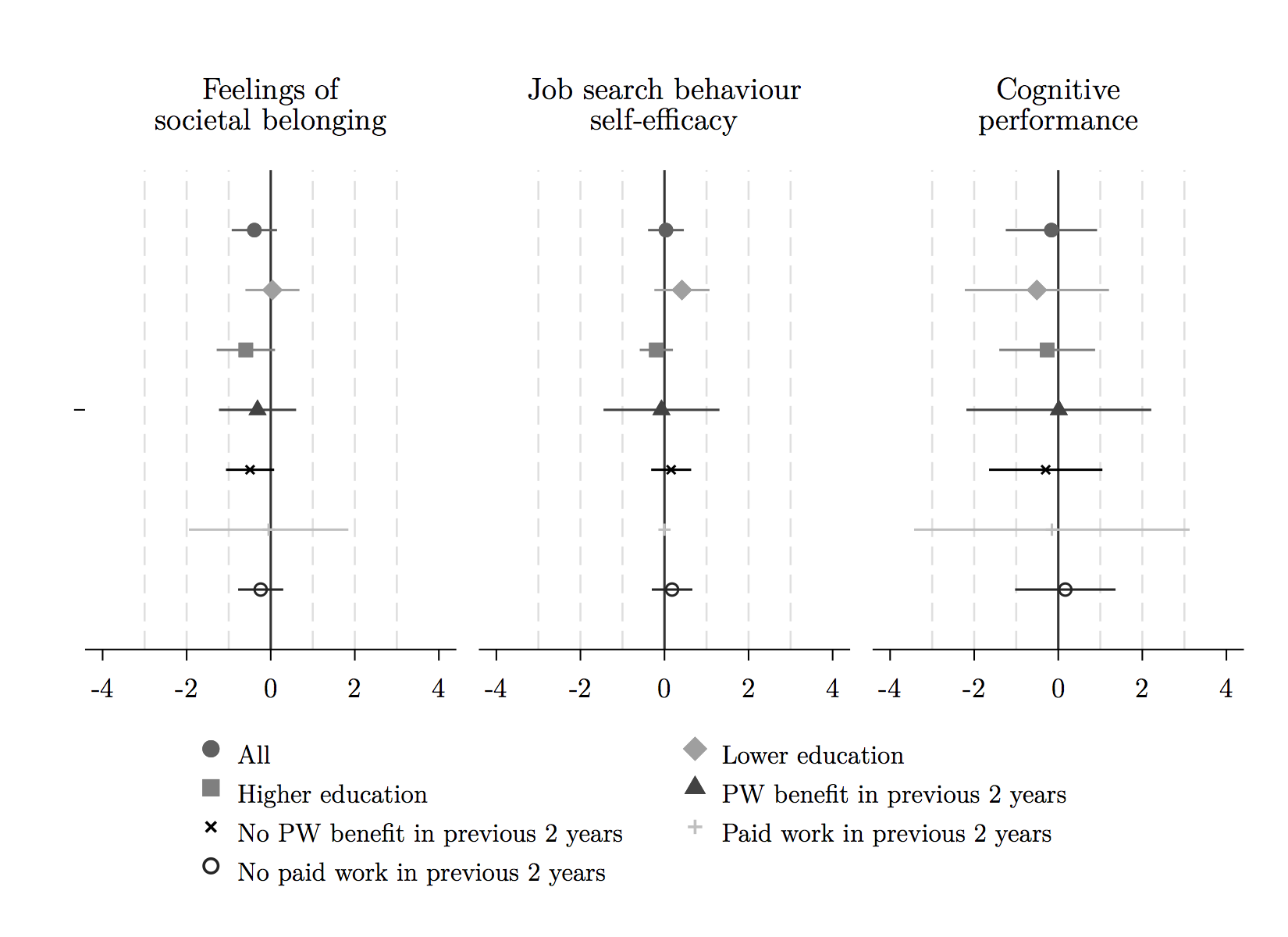

Supplement: S4 Fig — Source: See Fig 3. (TIF) [file pone.0252268.s006.tif]
